# Correlation between hyperreflective foci and visual function testing in eyes with intermediate age-related macular degeneration

**DOI:** 10.1186/s40942-023-00461-0

**Published:** 2023-04-07

**Authors:** T. Y. Alvin Liu, Jiangxia Wang, Karl G. Csaky

**Affiliations:** 1 Wilmer Eye Institute, Johns Hopkins University, Baltimore, MD USA; 2 Bloomberg School of Public Health, Johns Hopkins University, Baltimore, MD USA; 3 Retina Foundation of the Southwest, 9600 N Central Expy #200, Dallas, TX 75231 USA

**Keywords:** Age-related macular degeneration, Hyperreflective foci, Visual function, Cone function, Quantitative contrast sensitivity function, Low luminance, Microperimetry

## Abstract

**Background:**

To investigate the relationship between intraretinal hyperreflective foci (HRF) and visual function in intermediate age-related macular degeneration (iAMD).

**Methods:**

Retrospective, cross-sectional study. iAMD patients underwent spectral domain optical coherence tomography (SD-OCT) imaging and vision function testing: normal luminance best corrected visual acuity (VA), low luminance VA (LLVA), quantitative contrast sensitivity function (qCSF), low luminance qCSF (LLqCSF), and mesopic microperimetry. Each OCT volume was graded for the presence and number of HRF. Each HRF was graded for: separation from the retinal pigment epithelium (RPE), above drusen, and shadowing. Central drusen volume was calculated by the built-in functionality of the commercial OCT software after manual segmentation of the RPE and Bruch’s membrane.

**Results:**

HRF group: 11 eyes; 9 patients; mean age 75.7 years. No-HRF group: 11 eyes; 10 patients; mean age 74.8 years. In linear mixed effect model adjusting for cube-root transformed drusen volume, HRF group showed statistically significant worse VA, LLVA, LLqCSF, and microperimetry. HRF group showed worse cone function, as measured by our pre-defined multicomponent endpoint, incorporating LLVA, LLqCSF and microperimetry (p = 0.018). For eyes with HRF, # of HRF did not correlate with any functional measures; however, % of HRF separated from RPE and # of HRF that created shadowing were statistically associated with low luminance deficit (LLD).

**Conclusions:**

The association between the presence of HRF and worse cone visual function supports the hypothesis that eyes with HRF have more advanced disease.

## Background

Age related macular degeneration (AMD) is the leading cause of central vision loss in Americans over the age of 50. [1] Based on findings from the Age-Related Eye Disease Study (AREDS), eyes with large drusen and pigmentary abnormalities, as found on color fundus photographs, are more likely to develop advanced disease [2]. The advent of optical coherence tomography (OCT) has allowed for visualization of pathologies at a micron level. As such, in recent years, there has been a shift in focus to stratify AMD progression risk based on OCT features. The Classification of Atrophy Meeting (CAM) group, a group of multidisciplinary experts in retinal imaging, clinical trials and pathology, has identified OCT markers that are predictive of AMD progression, such as central drusen volume, subretinal drusenoid deposits (SDD) and hyperreflective foci (HRF). [3].

In parallel to the advancement of our understanding of OCT pathologies seen in AMD, it is also known that while patients with non-neovascular AMD can measure relatively well in visual acuity (VA) tests under normal luminance, they can exhibit significant deficits in other visual functional tests. For example, patients with non-neovascular AMD, as compared to normal controls, can have equivalent normal luminance BCVA, but worse visual functions, as measured by low luminance (LL) VA, microperimetry, dark adaptation rod function, [4] and mesopic high-contrast and low-contrast VA [5]. In addition, it’s been shown that the same cohort of intermediate AMD (iAMD) patients over a 12-month period can exhibit stable BCVA, with concurrent decline in microperimetry sensitivities [6]. Understanding the relationship between OCT biomarkers and various functional measures is critical for designing clinical trials that aim to halt or retard the progression of AMD, [7] as OCT biomarkers should only be used as surrogate endpoints if they are robustly correlated with meaningful primary functional endpoints.

In recent years, several studies have attempted to correlate OCT pathological features with visual function testing in AMD, but the results have been inconclusive. Neely et al. [8] found that in eyes with early AMD, dark adaptation was significantly delayed in eyes with SDD as compared to eyes without SDD, but the difference was no longer statistically significant after adjusting for age. In addition, Ou et al. [9] did not find a correlation between central drusen volume and low luminance deficit in eyes with iAMD. However, Ou et al. [10] did detect statistically significant differences between controls and intermediate/advanced (non-neovascular) AMD eyes in both normal and low luminance quantitative contrast sensitivity function (qCSF), as measured in area under log CSF (AULCSF).

As an extension of previous works, the current study aimed to correlate HRF, as detected on b-scan OCT images, with various visual function tests, including microperimetry, normal luminance VA, LLVA, qCSF and low luminance qCSF (LLqCSF). qCSF testing leverages active learning to allow for testing of contrast sensitivity at various combinations of spatial frequencies and contrast levels, whereas traditional Pelli-Robson charts only measure contrast at a single spatial frequency [11]. qCSF has been used to quantify visual function in other retinal diseases, such as diabetic retinopathy [12] and inherited retinal dystrophies [13]. Previous studies [4, 14] investigated the correlation between multiple visual function outcomes and non-neovascular AMD, but did not include qCSF. The underlying premise in this study is that the presence of HRF represents a more advanced disease state and is correlated with worse visual function, including qCSF, and with worse cone function, as measured by a multicomponent endpoint [15]. We chose to incorporate LLVA, LLqCSF and mesopic microperimetry into this multicomponent endpoint *a priori*, as all three outcomes measure to some degree cone function and can be considered collectively.

## Methods

Our study was a retrospective cross-sectional study that was approved by the University of Texas Southwestern (Dallas, TX) institutional review board. It adhered to the Declaration of Helsinki and the Health Insurance Portability and Accountability Act.

*Participants*. All study participants provided written informed consent, and were seen at the Retina Foundation of the Southwest (Dallas, TX). Inclusion criteria included: patients aged 55 years or older, iAMD as determined by retinal specialist (K.G.C) based on clinical examination and multimodal imaging, [16] and VA of 55 Early Treatment Diabetic Retinopathy Study (ETDRS) letters or more (Snellen 20/80 or better). Exclusion criteria included: presence of other significant retinal pathology, inability to complete study examinations, choroidal neovascularization, geographic atrophy on clinical examination, presence of incomplete (iRORA) or complete retinal pigment epithelium and outer retinal atrophy (cRORA) [17] on OCT, or presence of SDDs of > 9 disc areas (DA) in total or > 0.25 DA within 1 mm of the fovea [9]. iAMD patients with and without HRF on OCT imaging, as determined by two retinal specialists (K.G.C. and T. Y. A. L.) were included in our analysis. All participants underwent a comprehensive ophthalmic examination, including a dilated fundus examination and imaging with spectral domain optical coherence tomography (SD-OCT) [Spectralis, Heidelberg Engineering, Heidelberg, Germany], color fundus photography and fundus autofluorescence [Heidelberg Engineering, Heidelberg, Germany].

*Visual acuity testing and retinal imaging.* VA was tested under both normal and low-luminance conditions prior to imaging studies. VA was measured with spectacle correction using an ETDRS chart at 4 m at a luminance of 130 cd/m^2^, which was immediately followed by LLVA measurement that was performed by placing a 2.0-log unit neutral density filter over the study eye to reduce the luminance. Low luminance deficit (LLD) was defined as the difference between VA and LLVA measurements. SD-OCT imaging was obtained for each eye in high resolution mode, centering on the fovea with 97 horizontal B scans acquired over a scan area of 10 degrees vertical and 15 degrees horizontal. 30-degree fundus autofluorescence imaging centering on the fovea was performed for most eyes at the same visit.

*Macular Integrity Assessment (MAIA) Microperimetry.* Microperimetry tests were performed using the MAIA device [CenterVue, Padova, Italy], which uses a line-scanning laser ophthalmoscope for fundus tracking at 25 frames per second. Visual sensitivity thresholds at each location were measured using a 4 − 2 staircase strategy. A customized stimulus pattern consisting of Goldman spot size III at 37 locations and sampling the central 6º radius of the macular region was used. Microperimetry results were obtained on the same date as other vision function assessments, and involved a minimum of 2 prior baseline assessments. All MAIA results were required to be reliable, defined as having a false-positive rate of ≤ 25%. False-positive rates were determined based on the percentage of positive responses to presentations of suprathreshold stimuli to the optic nerve head (manually located before presenting the first stimulus). The microperimetry parameter examined in this study was the mean of the pointwise sensitivity results. All but three eyes underwent MAIA mesopic microperimetry testing.

*Contrast sensitivity testing*. qCSF testing was performed with spectacle correction at 3 m in a dimly-lit room, under both standard luminance and low luminance (without dark adaptation and placing a 2.0-log unit neutral density filter over the study eye). Each patient was presented with a series of 25 triplets (75 optotypes of various sizes and contrasts), and asked to identify the letters on a NEC LED monitor (Manifold Contrast Vision Meter) with a screen size of 19.4 × 11.0 degrees of visual angle and a background luminance of 90 cd/ m^2^ [Adaptive Sensory Technology, San Diego, CA]. The optotypes used were special bandpass-filtered Sloan optotypes, and the optotypes for each round of testing were chosen from a wide range of contrast levels (Michelson contrast 0.20 − 98.20%) and spatial frequencies (1.0–27 cpd) to maximize the information gained [18]. The patients’ responses for each optotype (correct, incorrect or unknown) were recorded. For each eye, the contrast sensitivity values (in Log CS) were plotted against spatial frequencies, creating a log CSF curve. The area under log CSF (AULCSF) was obtained at both standard and low luminance for each eye, and the low luminance deficit AULCSF (LLD AULCSF) was calculated as the difference between the two values.

**Fig. 1:**
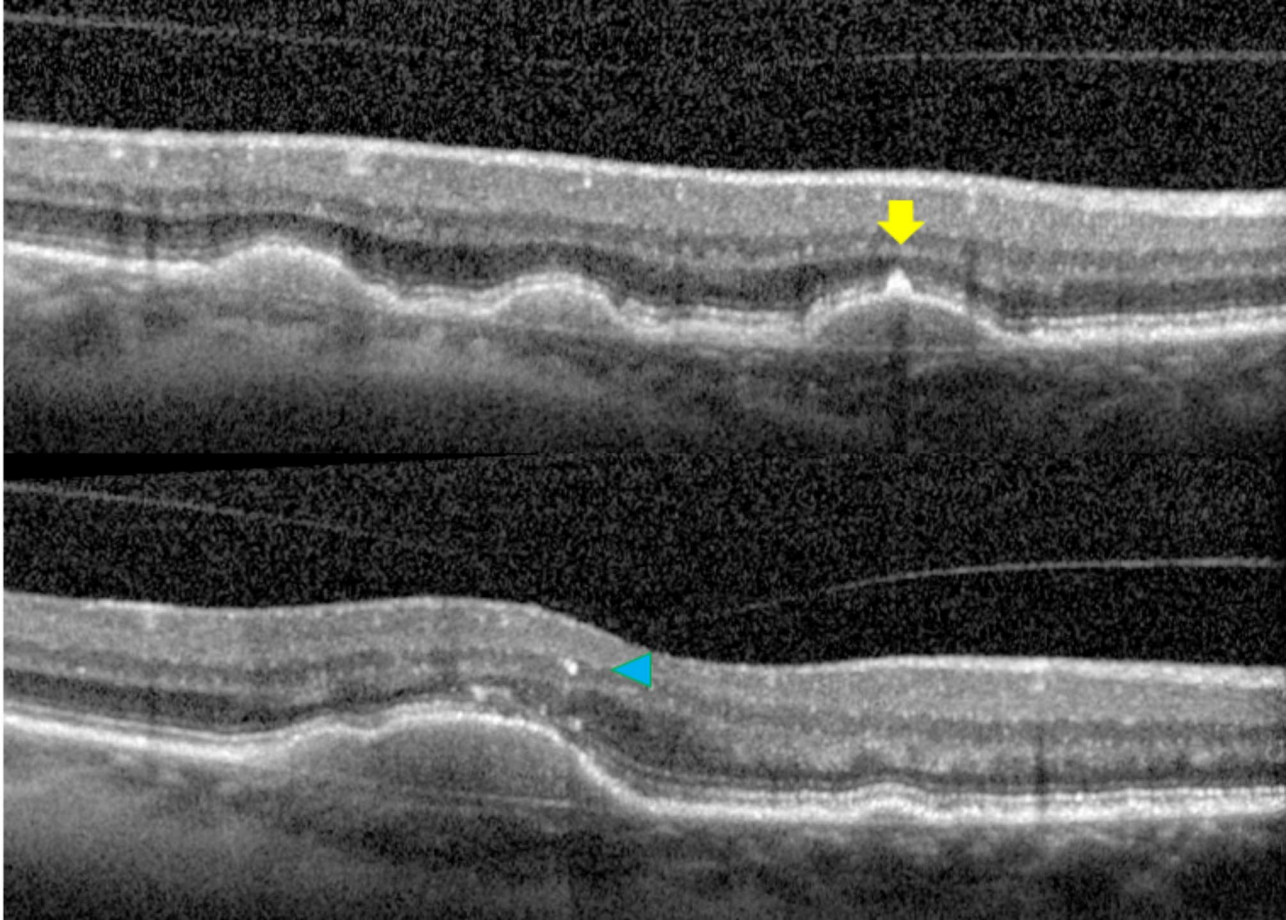
Sample hyperreflective focus that is attached to the retinal pigment epithelium (yellow arrow, top panel) and one that has migrated anteriorly to the level of inner nuclear layer (blue arrow head, bottom panel)

**Fig. 2:**
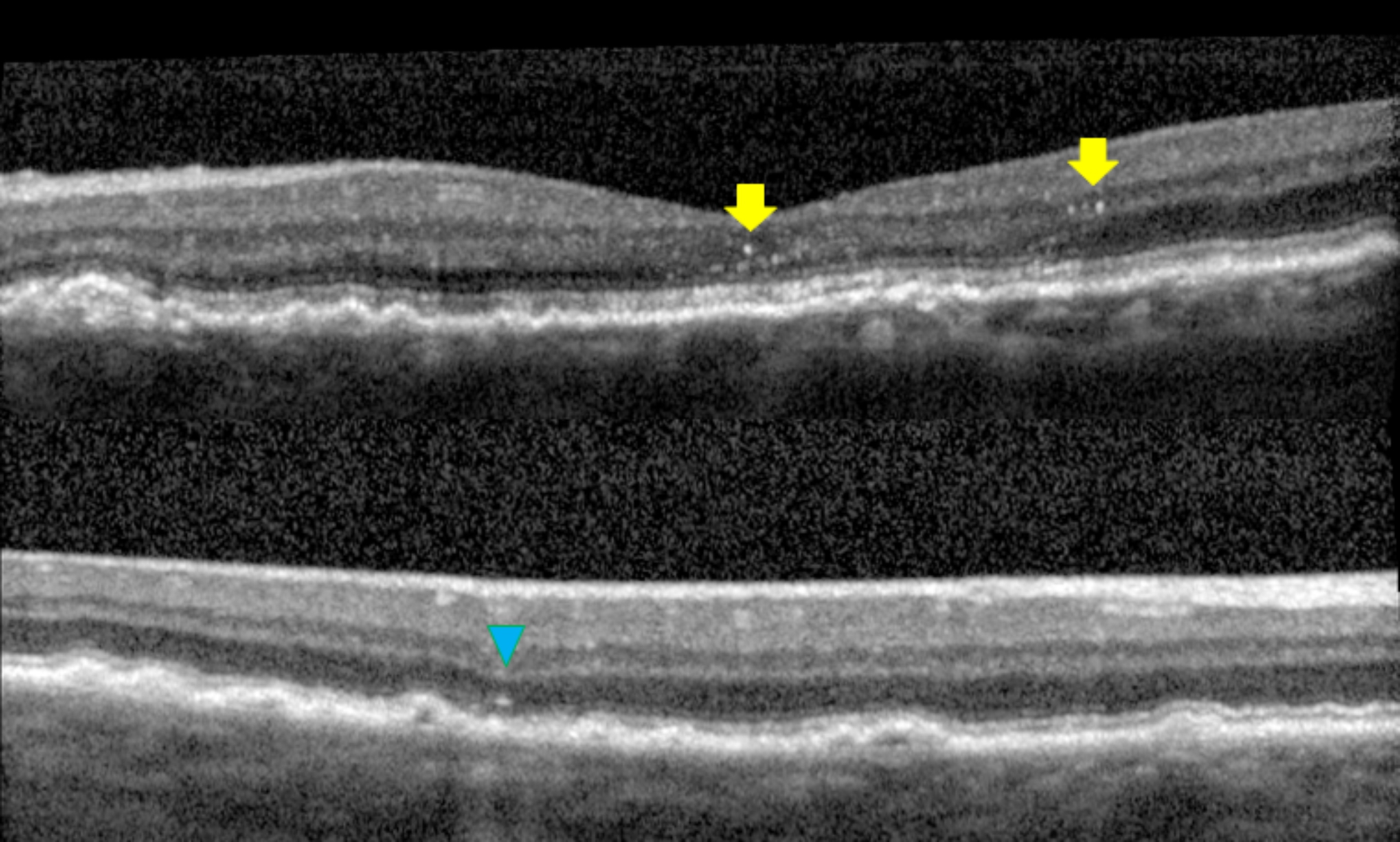
Sample hyperreflective flecks, but not foci, as they do not meet the 3-pixel threshold (yellow arrows, top panel). While most hyperreflective foci are above drusen, this is a HRF that is not directly above a druse (blue arrowhead, bottom panel)

**Fig. 3:**
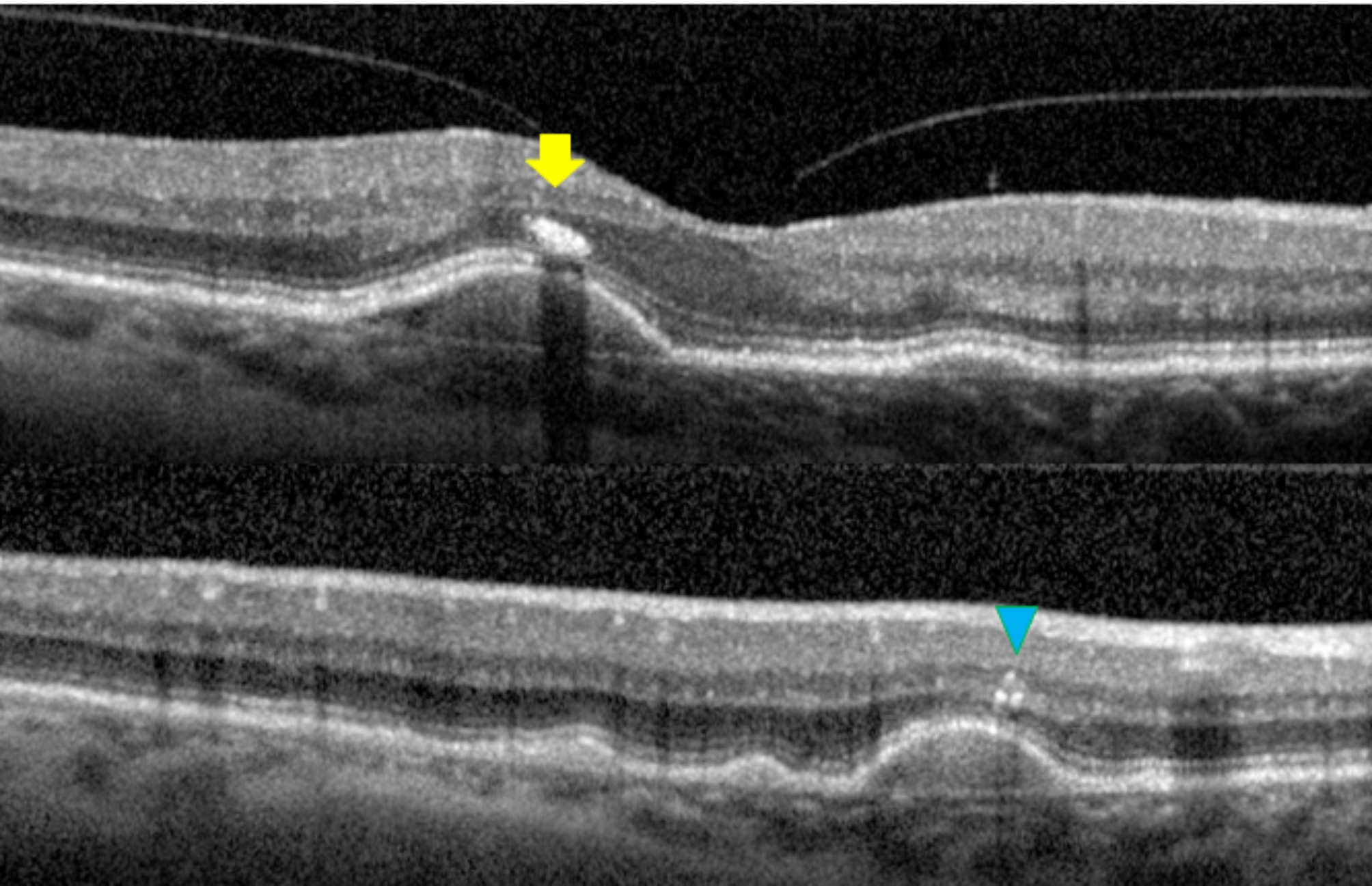
A single, large hyperreflective focus with intense shadowing (yellow arrow, top panel). A cluster of three hyerreflective foci separated internally by hyporeflectivity (blue arrowhead, bottom panel)

*Hyperreflective foci and central drusen volume grading*. Each SD-OCT volume was graded by a board-certified retinal specialist (T.Y.A.L.) for the number of hyperreflective foci seen within the scanned volume. A hyperreflective focus by definition has a reflectivity similar to or more intense than that of the retinal pigment epithelium (RPE)-Bruch’s membrane band and is at least 3-pixel wide [19] as measured in Image J [National Institute of Health, Bethesda, USA]. A cluster of candidate HRF was counted as a single HRF, if the cluster was not separated internally by hyporeflectivity at auto magnification [14]. Large but dim HRF candidates were counted in our analysis, as it is known that cells could lose granules as they migrate anteriorly towards the inner retina [20]. In addition, each HRF was graded for the following four variables: innermost retinal layer to which the HRF extended, whether it was separated from the RPE (Yes vs. No), whether it was above drusen (Yes vs. No), and whether there was shadowing of the underlying structures (Yes vs. No). The spectrum of HRF included in our study was shown in Figs. 1, 2 and 3. The RPE and Bruch’s membrane was manually segmented by T.Y.A.L. in each b-scan. Drusen volume was defined as the volume between the inner border of the RPE and Bruch’s membrane, and the central 3 mm drusen volume of each eye was calculated by the built-in functionality of the commercial Spectralis Heidelberg OCT software.

*Statistical analysis*. Linear mixed effects models, adjusting for cube-root transformed drusen volume and VA, were used to compare the 7 visual function outcomes between the HRF and no-HRF groups. A random intercept for patients was included in the models to account for the correlation between OD and OS of bilateral patients. The multicomponent endpoint, combining LLVA, LLqCSF and average mesopic MAIA threshold, was also compared between the two groups, using the nonparametric O’Brien’s test. To quantify the spread of signal intensities within each eye recorded across the 37 targets on MAIA microperimetry, mean and standard deviation (SD) of signal intensity for each eye was calculated. The mean and SD of MAIA microperimetry signal intensities of the HRF group and no-HRF group were compared, using linear mixed effects model. For the HRF group, Spearman’s correlation coefficient was used to examine the correlation between the number of HRFs and the functional metrics within each eye. Considering potential correlation between the eyes from the same patient, a sensitivity analysis of using one eye per patient was also carried out. Spearman’s correlation coefficient was used to examine the correlation between the 4 qualitative variables of HRF and the functional metrics within each eye. All statistical analysis was performed using the Stata v17.0 software.

## Results

In total, 11 eyes from 9 patients and 11 eyes from 10 patients were included in the HRF and no-HRF group, respectively. The mean age was 75.7 and 74.8 years for the HRF and no-HRF group, respectively (p = 0.83). 89% and 40% patients were female in HRF and no-HRF group, respectively. All patients were Caucasian, and there was no difference in age between the groups. There was a difference in the mean central 3 mm drusen volume between the HRF group (0.3 mm [3]) and no-HRF group (0.12 mm [3]) (p = < 0.001). The mean number of HRF (SD) for each eye with HRF was 24 (±15). (Table 1)

**Table 1:** Patient and eye characteristics by HRF status

Factor	No-HRF	HRF	All	p-value
**Patient-level**				
N (Patients)	10	9	19	
Age at visit (years), mean (SD)	74.8 (10.2)	75.7 (6.4)	75.2 (8.4)	0.83
Female Gender, N (%)	4 (40%)	8 (89%)	12 (63%)	0.027
White Race/ ethnicity, N (%)	10 (100%)	9 (100%)	19 (100%)	
**Eye-level**				
N (Eyes)	11	11	22	
Drusen volume mm^3^, mean (SE)	0.12 (0.04)	0.30 (0.03)	0.20 (0.03)	< 0.001

HRF = hyerreflective foci. SD = Standard Deviation. SE = Standard Error

As compared to the no-HRF group, the HRF group had worse measurements in multiple visual function metrics.

In linear mixed effect analysis adjusting for cube-root transformed drusen volume (Table 2), the following visual function metrics remained statistically different between HRF and no-HRF groups: VA, LLVA, LLqCSF, LLDqCSF and average MAIA threshold. Secondary linear mixed effect analysis, after adjusting for the presence and absence of HRF, demonstrated that cube-root transformed drusen volume was associated with VA and LLD.

**Table 2:** Linear mixed effects models comparing the visual function metrics between the HRF and no-HRF group

Metric	Adjusting for cube-root transformed drusen volume			Adjusting for VA		
	HRF: yes vs. no	95% CI	P value	HRF: yes vs. no	95% CI	P value
**VA**	-9.36	-16.52, -2.19	**0.011**	-	-	**-**
**LLVA**	-9.88	19.40, -0.36	**0.042**	-7.24	-12.0, -2.44	**0.003**
**LLD**	1.35	-4.71, 7.42	0.662	7.24	2.44, 12.04	**0.003**
**qCSF**	-0.19	-0.48, 0.11	0.215	-0.15	-0.30, 0.00	0.054
**LLqCSF**	-0.2	-0.34, -0.06	**0.005**	-0.08	-0.15, -0.01	**0.023**
**LLDqCSF**	-0.26	-0.49, -0.04	**0.023**	-0.05	-0.16, 0.06	0.399
**MAIA**	-4.08	-8.14, -0.02	**0.049**	-3.05	-5.72, -0.39	**0.025**

HRF = hyperreflective foci. VA = visual acuity. LLVA = low luminance visual acuity. LLD = low luminance deficit. qCSF = quantitative contrast sensitivity function. LLqCSF = low luminance quantitative contrast sensitivity function. LLDqCSF = low luminance deficit quantitative contrast sensitivity function. MAIA = average MAIA perimetry sensitivity in dB. P values in bold are statistically significant

In linear mixed effect analysis adjusting for VA (Table 2), the following visual function metrics remained statistically different between HRF and no-HRF groups: LLVA, LLD, LLqCSF and average mesopic MAIA threshold. Secondary linear mixed effect analysis, after adjusting for the presence and absence of HRF, demonstrated that VA was associated with all outcomes except for LLD.

The HRF group also showed worse cone function, as measured by our a-priori-defined multicomponent endpoint that combines LLVA, LLqCSF and average mesopic MAIA threshold (p = 0.018 from the nonparametric O’Brien’s test).

The signal intensity variation on MAIA testing within each eye was compared between the HRF and no-HRF group, using linear mixed effects models. The HRF group showed higher intra-eye variations (p = 0.004) and lower mean signal intensity (p = 0.016).

We performed additional analyses for the HRF group. No significant correlation between the number of HRF and the 7 functional measures was observed. Each HRF was also graded qualitatively on whether it was separated from the RPE, above drusen and created shadowing of the underlying structures. The % of HRF separated from the RPE and total number of HRF that created shadowing were statistically associated with LLD.

## Discussion

Our study was carried out to interrogate the concept that patients with HRF represent a more advanced state of iAMD and, as such, these patients will exhibit worse visual function, when compared to iAMD patients without HRF. Indeed, our data demonstrated that eyes with HRF, as compared to eyes without HRF, exhibited worse cone function. Not only did eyes with HRF showed worse VA, LLVA, LLqCSF and average mesopic MAIA threshold, they also performed worse on our pre-defined multicomponent end point. We chose to include LLVA, LLqCSF and mesopic MAIA in our multicomponent endpoint *a priori*, as all three measure cone function to varying degrees.

Coincidentally, our HRF group also had a higher mean central drusen volume. In our secondary analysis adjusting for the presence and absence of HRF, cube-root transformed central drusen volume was found to be associated with LLD measurement, which was in contrast to our prior study showing no such correlation in eyes with iAMD [9]. In our previous study, the presence of HRF was not evaluated, so it was possible that the lack of adjustment for HRF accounted for the negative finding.

In our study, eyes with HRF had both lower mean MAIA threshold values and higher intra-eye signal intensity variation. Again, the presence of HRF appears to indicate more diffuse, advanced disease that involves localized damage and dysfunction within the cone cells, leading to more variable signal intensity measurements. However, within the HRF group, a higher number of HRF within each eye did not correlate with any of the 7 functional measures: VA, LLVA, LLD, qCSF, LLqCSF, LLDqCSF and average MAIA threshold. This is either because our study was underpowered to detect a difference or the number of HRF is not an accurate surrogate marker for disease severity. Perhaps, the HRF area would be a more robust predictor for worse visual function, as HRF area has been shown to be significantly correlated with progression to late AMD over the course of one year [21]. Of note, we showed that certain qualitative features of HRF, such as separation from RPE, were associated with certain functional outcomes, such as LLD. This could be explained by the natural history of HRF. HRF form at the level of the RPE and gradually migrate anteriorly towards the inner retina. HRF that are completely separated from the RPE, as compared to HRF that are still attached to the RPE, are likely later in their life cycle and could be surrogate markers for RPE dysfunction for a longer duration of time.

A recent study by Echos et al. [14] demonstrated that HRF were associated with delayed rod mediated dark adaptation but less strongly with cone medicated vision. Two major differences exist between their study and our study. Their study included normal patients, patients with early AMD and patients with iAMD, while our study only included patients with iAMD. Their study used Pelli-Robson charts that can only measure contrast sensitivity at a single spatial frequency, while our study utilized more sophisticated qCSF techniques that can measure contrast sensitivity at various combinations of spatial frequencies and contrast levels. Our study contributes to the existing literature by confirming that HRF are also strongly associated with cone mediated function and the associated decline in cone function can even be discerned among AMD patients with the same stage of disease (iAMD). Our study provides another strong line of evidence to support HRF being used as a structural endpoint in clinical trials that aim to prevent the progression from iAMD to advanced AMD.

## Conclusions

Our study demonstrated an association between the presence of HRF with cone mediated visual function in eyes with iAMD, thus further supporting the usefulness of using HRF as a surrogate marker for disease severity. However, our study is limited by the small sample size and its cross sectional nature. As the next step, a larger study including patients with both early and iAMD will be conducted. Longitudinal imaging and functional data will be collected to investigate whether longitudinal structural changes, such as the change in HRF area, are correlated with change in visual function.

## Data Availability

Request for access to the original data can be made to the corresponding author.

## Abbreviations

HRF: hyperreflective foci
iAMD: intermediate age-related macular degeneration
SD-OCT: spectral domain optical coherence tomography
VA: visual acuity
LLVA: low luminance VA
qCSF: quantitative contrast sensitivity function
LLqCSF: low luminance qCSF
RPE: retinal pigment epithelium
AREDS: Age-Related Eye Disease Study
CAM: Classification of Atrophy Meeting
SDD: subretinal drusenoid deposits
AULCSF: area under log CSF
ETDRS: Early Treatment Diabetic Retinopathy Study
cRORA: complete retinal pigment epithelium and outer retinal atrophy
DA: disc areas
LLD: low luminance deficit
MAIA: Macular Integrity Assessment
LLD AULCSF: low luminance deficit AULCSF
SD: standard deviation
